# Response to inhibition of smoothened in diverse epithelial cancer cells that lack smoothened or patched 1 mutations

**DOI:** 10.3892/ijo.2012.1599

**Published:** 2012-08-22

**Authors:** FABRIZIO GALIMBERTI, ALEXANDER M. BUSCH, FADZAI CHINYENGETERE, TIAN MA, DAVID SEKULA, VINCENT A. MEMOLI, KONSTANTIN H. DRAGNEV, FANG LIU, KEVIN C. JOHNSON, YONGLI GUO, SARAH J. FREEMANTLE, ANGELINE S. ANDREW, PATRICIA GRENINGER, DAVID J. ROBBINS, JEFF SETTLEMAN, CYRIL BENES, ETHAN DMITROVSKY

**Affiliations:** 1 Departments of Pharmacology and Toxicology; 2 Pathology; 3 Community and Family Medicine; 4 Medicine, and; 5 The Norris Cotton Cancer Center, Geisel School of Medicine at Dartmouth, Hanover, NH 03755 and Dartmouth-Hitchcock Medical Center, Lebanon, NH 03756;; 6 Center for Molecular Therapeutics, Massachusetts General Hospital Cancer Center, Charlestown, MA 02129, USA

**Keywords:** hedgehog, smoothened, patched, lung cancer

## Abstract

Hedgehog (HH) pathway Smoothened (Smo) inhibitors are active against Gorlin syndrome-associated basal cell carcinoma (BCC) and medulloblastoma where Patched (Ptch) mutations occur. We interrogated 705 epithelial cancer cell lines for growth response to the Smo inhibitor cyclopamine and for expressed HH pathway-regulated species in a linked genetic database. Ptch and Smo mutations that respectively conferred Smo inhibitor response or resistance were undetected. Previous studies revealed HH pathway activation in lung cancers. Therefore, findings were validated using lung cancer cell lines, transgenic and transplantable murine lung cancer models, and human normal-malignant lung tissue arrays in addition to testing other Smo inhibitors. Cyclopamine sensitivity most significantly correlated with high cyclin E (P=0.000009) and low insulin-like growth factor binding protein 6 (IGFBP6) (P=0.000004) levels. Gli family members were associated with response. Cyclopamine resistance occurred with high GILZ (P=0.002) expression. Newer Smo inhibitors exhibited a pattern of sensitivity similar to cyclopamine. Gain of cyclin E or loss of IGFBP6 in lung cancer cells significantly increased Smo inhibitor response. Cyclin E-driven transgenic lung cancers expressed a gene profile implicating HH pathway activation. Cyclopamine treatment significantly reduced proliferation of murine and human lung cancers. Smo inhibition reduced lung cancer formation in a syngeneic mouse model. In human normal-malignant lung tissue arrays cyclin E, IGFBP6, Gli1 and GILZ were each differentially expressed. Together, these findings indicate that Smo inhibitors should be considered in cancers beyond those with activating HH pathway mutations. This includes tumors that express genes indicating basal HH pathway activation.

## Introduction

Hedgehog (HH) signaling is important in development and carcinogenesis because it controls cell fate and proliferation (1,2). Three mammalian HH ligands [sonic (sHH), desert, and indian] are lipid-modified secreted proteins (1,2). The HH receptor Patched 1 (Ptch1) inhibits this pathway by locking Smoothened (Smo) in an inactive conformation. After HH ligand treatment, Ptch1 releases Smo inhibition, which augments Gli family member (Gli1, Gli2, and Gli3) expression (1,2). Gli1 and Gli2 typically exert stimulatory while Gli3 has inhibitory effects (1). Activating the HH pathway affects expression of Ptch1, cyclin D1, cyclin E, insulin-like growth factor 2 (IGF2), insulin-like growth factor binding protein 6 (IGFBP6), GILZ and other species (2–5).

HH pathway deregulation occurs in many tumors including lung (6), breast (7), and pancreatic (8) cancers. Ptch1 mutations occur in the Gorlin syndrome-associated cancers basal cell carcinoma (BCC) and medulloblastoma (9–12). Smo inhibition can chemoprevent invasive BCC (13). Cyclopamine, a naturally-occurring HH antagonist, binds to Smo and inhibits HH signaling (14). Other Smo inhibitors exist with antineoplastic effects *in vitro* and in clinical trials for patients with BCC or medulloblastoma (9–12). The HH pathway regulates growth of small cell lung cancer (SCLC) and non-small cell lung cancer (NSCLC) (6,15). HH pathway members are abundantly expressed in the premalignant and malignant lungs of cyclin E-expressing transgenic mice (16). Resistance to Smo inhibitors occurs with acquired Smo mutations (17,18).

This study uncovered growth inhibitory responses to Smo inhibition in diverse cancer cells using a robotic-based platform with a genetic database. In this database Ptch1 and Smo sequences were available with information about expression of species associated with HH pathway activation. Basal expression of these species in cancer cells was hypothesized to indicate growth dependence of these cells on the HH pathway. It was hypothesized that cancer cells expressing these species would respond to a Smo inhibitor.

Multiple Smo inhibitors were studied in lung cancer because the HH pathway is active in subsets of these cancers. Both murine and human lung cancer cell lines exist. Cyclin E-driven transgenic and transplantable murine lung cancer models that spontaneously activated the HH pathway were available for study as was a paired human normal-malignant lung tissue array with an associated clinical database. The presented findings implicate use of Smo inhibitors for lung and other cancers when a gene profile indicative of HH pathway dependence is expressed in the cancer cells.

## Materials and methods

### Cell culture

ED-1 and ED-2 murine lung cancer lines, C-10 murine immortalized lung epithelial cells, BEAS-2B human immortalized bronchial epithelial cells, and human lung cancer cell lines (A549, HOP-62, H-522, U-1752, NCI-H1730, and NCI-H2122) were each cultured in RPMI-1640 medium with 10% fetal bovine serum (FBS) and 1% antibiotic and antimycotic solution at 37°C in 5% CO_2_ in a humidified incubator, as before (15,16,19–21). Cell lines were obtained from and authenticated (using genotypic and phenotypic assays) by ATCC except for murine ED-1 and ED-2 lung cancer cell lines that were previously described and authenticated (19,21).

### Chemicals

Cyclopamine (LC Laboratories, Wobrun, MA) and tomatidine (Sigma-Aldrich, St. Louis, MO) were purchased as were recombinant mouse sHH (R&D Systems, Minneapolis, MN) and FBS (Gemini Bioproducts, Inc, Calabasas, CA). The Smo inhibitor MK-4101 (22) was provided by Merck. The SANT-1 Smo inhibitor (15) was purchased (Tocris Bioscience, Ellisville, MO) as was the SAG Smo agonist (EMD Millipore, Billerica, MA).

### Repression of HH pathway members

Cells were independently treated with the Smo inhibitors: cyclopamine, SANT-1 and MK-4101. *In vivo* Smo inhibition was achieved in mouse lung cancer models with cyclopamine (intraperitoneal injections, 40 mg/kg) treatments or with short hairpin RNA (shRNA)-mediated Smo knock-down in ED-1 cells. Individual small interfering RNA (siRNA)-mediated or shRNA-mediated repression of Gli1, Gli2, or Gli3 was achieved.

### High-throughput proliferation assays

Cyclopamine growth effects were investigated in 705 human cancer cell lines using a high-throughput screen (19,23,24). Cells were treated with cyclopamine at 10 μM (and lower dosages) in media with 5% FBS and were assayed at 72 h with quantification by the SpectraMax M5 plate reader (Molecular Devices, Sunnyvale, CA). Means of triplicate cyclopamine treatment experiments were compared to vehicle controls, using optimized methods (19,23,24).

### Smo inhibitor responses

The HH pathway affects expression of Ptch1, cyclin D1, cyclin E, IGF2, IGFBP6, GILZ, Gli family members, and other species (2–5). The cor.test function (25) of R (26) compared cyclopamine-dependent growth responses to expressed species. Expression values were from U133 Plus 2.0 Affymetrix arrays and are publically available (27). The data set consisted of 490 samples corresponding to 164 unique cell lines that were typically examined in triplicate. Correlations were done: a) using all samples and b) using samples with a cyclopamine growth inhibitory response ≤0.75 at the 10 μM dosage, corresponding to 110 samples (in triplicate). Cyclopamine-mediated growth response was compared with mutation data available from the Sanger database (28).

### Ptch1 and smo sequence analyses

Sequencing of the coding regions of Ptch1 was performed as before (29). For murine cell lines, murine-specific primers were used to sequence homologous domains of Ptch1. To assess for Smo mutations in murine and human cell lines, prior mutations in human Smo were searched for (A324T, V404M, D473H, E518K, W535L, and T640A) and corresponding regions of murine Smo were sequenced.

### Transient transfection, proliferation and apoptosis assays

Logarithmically growing ED-1 (3×10^4^ and 4.5×10^3^), ED-2 (8×10^4^), C-10 (1×10^5^), BEAS-2B (3×10^5^), A549 (5×10^4^), HOP-62 (5×10^4^), H-522 (3×10^5^), U1752 (5×10^4^), NCI-H1703 (4.5×10^5^), and NCI-H2122 (6×10^5^) cells were individually plated onto each well of 6-well tissue culture plates (BD Bioscience, San Jose, CA) 24 h before transfection or drug treatments. Three replicate experiments were performed in triplicate. Logarithmically growing cells were assayed using the CellTiter-Glo assay (Promega, Madison, WI) (21). Apoptosis was scored with the Caspase-Glo 3/7 Assay System (Promega).

SiRNA transfections were with Oligofectamine reagent (Invitrogen, Carlsbad, CA). SiRNAs targeting Gli1, Gli2, Gli3, Smo, IGFBP6 or a RISC-free control were each purchased (Dharmacon, Lafayette, CO). Two different siRNAs independently targeted Gli1, Gli2, Gli3, Smo, or IGFBP6 sequences: 5′-GGUUGGAACUUCUGUGAUG-3′ (Gli1.1), 5′-GAGCAGGCCUCCGUUGUA-3′ (Gli1.2), 5′-GGGAGAAGAAGGAGUUCGU-3′ (Gli2.1), 5′-GGUUUGUGGUUGAGCGGAA-3′ (Gli2.2), 5′-CCAUCGGUGGAAAAGCGU-3′ (Gli3.1), 5′-GAAACGCAAUCACUAUGCA-3′ (Gli3.2), 5′-AGAACCCGCUGUUCACCGA-3′ (Smo1.1), 5′-GCAUCUGUUUUGUAGGCUA-3′ (Smo1.2), 5′-CAUCGAGGCUUCUACCGGA-3′ (IGFBP6-1), and 5′-CAACAGAGGAAUCCAGGCA-3′ (IGFBP6-2). Transient transfections were with FuGENE 6 reagent (Roche Applied Science, Indianapolis, IN). Reporter assays were performed after transfection of a GLIBS-Luciferase reporter construct or TK-Luciferase control plasmid (19). The Dual-Luciferase reporter assay system (Promega) was used in three replicate experiments, each performed in triplicate.

### Stable knock-down

ED-1 cells stably expressing green fluorescent protein (GFP) were individually infected with lentivirus expressing shRNAs targeting Smo: TRCN0000026245 (Smo1) and TRCN0000026295 (Smo2); IGFBP6: TRCN0000114766 (IGFBP6.1) and TRCN0000114768 (IGFBP6.2); GILZ: TRCN0000085743 (GILZ.1) and TRCN0000085746 (GILZ.2), or a scrambled control shRNA (RHS4080) (Open Biosystems, Huntsville, AL). Selection was with puromycin (5 μg/ml). Knock-downs were determined using semi-quantitative real-time reverse transcription (RT) polymerase chain reaction (PCR) assays. Cells with the most robust Smo knock-down versus controls were selected for tail vein injection experiments into syngeneic mice (19,20,30).

### Real-time RT-PCR assays

Total RNA was isolated using the RNeasy kit (Qiagen, Valencia, CA). RT was with the High Capacity cDNA RT kit (Applied Biosystems, Foster City, CA) and a Peltier Thermal Cycler (MJ Research, Waltham, MA). Semi-quantitative real-time RT-PCR assays were with SYBR Green PCR Mastermix (Applied Biosystems) and the 7500 Fast real-time PCR system (Applied Biosystems). Human IGFBP6 and β-actin qPCR assays were performed with TaqMan Universal PCR Master Mix and the manufacturer’s described assays (Applied Biosystems). Three replicate experiments were performed. Primers were: murine Gli1 forward: 5′-CCAAGCCAACTTTATGTCAGGG-3′, and reverse: 5′-AGCCCGCTTCTTTGTTAATTTGA-3′; murine Gli2 forward: 5′-CAACGCCTACTCTCCCAGAC-3′, and reverse: 5′-GAGCCTTGATGTACTGTACCAC-3′; murine Gli3 forward: 5′-CACAGCTCTACGGCGACTG-3′, and reverse: 5′-CTGCATAGTGATTGCGTTTCTTC-3′; murine Smo forward: 5′-GAGCGTAGCTTCCGGGACTA-3′, and reverse: 5′-CTGGGCCGATTCTTGATCTCA-3′; murine IGFBP6 forward: 5′-TGCTAATGCTGTTGTTCGCTG-3′, and reverse: 5′-CACGGTTGTCCCTCTCTCCT-3′; murine GILZ forward: 5′-ACCACCTGATGTACGCTGTG-3′, and reverse: 5′-TCTGCTCCTTTAGGACCTCCA-3′; murine glyceraldehyde 3-phosphate dehydrogenase (GAPDH) forward: 5′-AGGTCGGTGTGAACGGATTTG-3′, and reverse: 5′-TGTAGACCATGTAGTTGAGGTCA-3′; human Gli1 forward: 5′-GGCACCATGAGCCCATCTC-3′, and reverse: 5′-ATCACCTTCCAAGGGTTCCTC-3′; human Gli2 forward: 5′-GGTGAAGCCTCCACCCTTTC-3′, and reverse: 5′-TGCATGTAGTTTACCCTGGGG-3′; human Gli3 forward: 5′-CTCCACGACCACTGAAAAGAAA-3′, and reverse: 5′-TCTCTGTGATAAGTCTGTCCAGG-3′; human Smo forward: 5′-GGCAACAGCATTGCAGTGAAG-3′, and reverse: 5′-GAGGAGAGACACACGAGCCT-3′; and human GAPDH forward: 5′-ATGGGGAAGGTGAAGGTCG-3′, and reverse: 5′-GGGGTCATTGATGGCAACAATA-3′.

### Clonal growth assays

Two hundred logarithmically growing ED-1 cells were plated for triplicate replicate clonal growth assays, as before (20). Colonies were stained with Diff Quick (IMEB Inc, San Marcos, CA) and were counted with the Oxford Optronix Col Count counter (Oxford Optronix, Oxford, UK) (31).

### Normal-malignant lung tissue array

The New Hampshire State Cancer Registry and the Dartmouth-Hitchcock Medical Center Tumor Registry have been described (20). Analyses of normal versus malignant lung tissue arrays were by a pathologist (Vincent A. Memoli) unaware of findings from the associated clinical database. Signed consents were obtained; studies were reviewed and approved by Dartmouth’s Institutional Review Board (IRB) for human subjects. A paired normal-malignant lung tissue microarray was constructed (20).

### Statistical analysis

Results were expressed as means ± standard deviations. Results of all independent experiments were pooled to assess for statistical significance. Z-test and two-sided t-tests were used for statistical analyses with significance considered for values of P<0.05.

### Cyclopamine treatments of transgenic mice

Three 9-month-old female mice expressing transgenic wild-type human cyclin E were each treated daily (intraperitoneal) for 5 consecutive days with cyclopamine (40 mg/kg) or vehicle (45% hydroxypropyl-ß-cyclodextrin, HBS) with a total of six mice studied. Mice were examined using an Institutional Animal Care and Use Committee (IACUC)-approved protocol. Tissues were formalin-fixed, paraffin-embedded and sectioned for histopathology (20). Hematoxylin and eosin staining as well as Ki-67, cyclin D1, and cyclin E immunostaining were performed as before (16,19). Analyses were by a pathologist (Vincent A. Memoli) unaware whether harvested tissues were from cyclopamine or vehicle-treated mice.

### Murine syngeneic lung cancer transplantation assays

ED-1 cells were transduced with a GFP expression vector and transductants were sorted (15,16) with 8×10^5^ cells injected into tail veins of each female FVB mouse (8-week old). Ten mice per arm were used and replicate experiments were performed. To investigate cyclopamine antineoplastic effects, 10 mice were each intraperitoneally treated daily for 14 days with cyclopamine (40 mg/kg); 10 additional mice were each treated with vehicle (45% HBS). Treatments began 2 weeks after tail-vein injections because lung tumors formed by then (data not shown). Ten syngeneic FVB mice were each injected with ED-1 cells that were treated with either MK-4101 (10 μM) or vehicle as part of an IACUC-approved protocol. Mice were sacrificed 28 days post-tail vein injections. Replicate experiments were performed. Lung tissues were formalin-fixed, paraffin-embedded and sectioned for histopathology (16,19,20). A rabbit polyclonal anti-GFP antibody (product ab290) (Abcam, Cambridge, MA) was used to identify lung tumors. Hematoxylin counterstaining was used.

## Results

### Growth effects of a Smo inhibitor were studied using a robotic-based platform linked to a genetic database (19,23,24)

**Figure 1.**
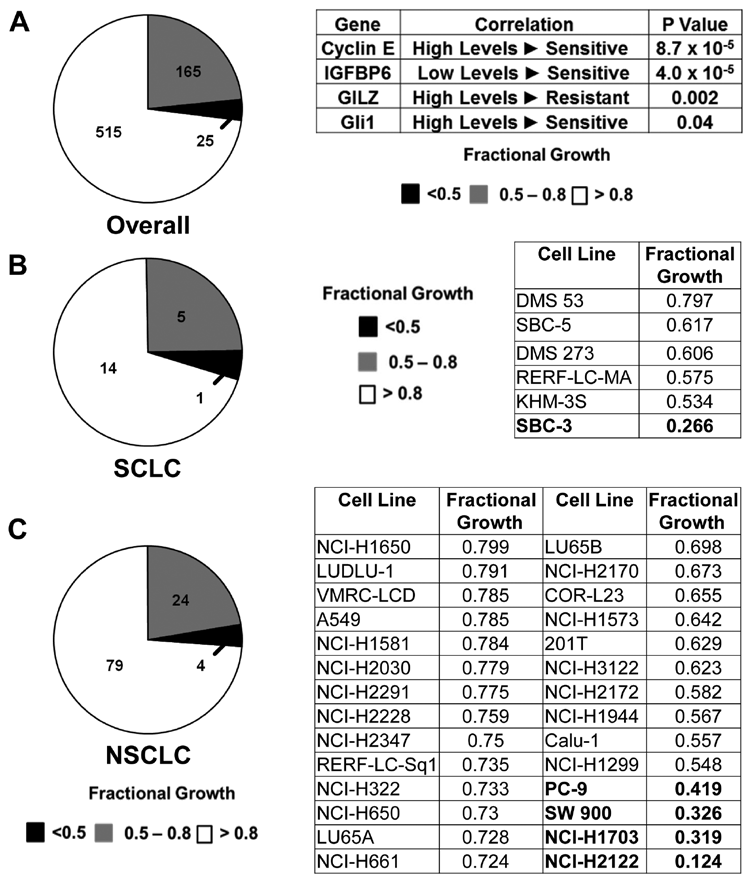
Cyclopamine-mediated growth inhibition of diverse human epithelial cancer cell lines. (A) Overall profile of cyclopamine (10 μM)-mediated growth response was interrogated in 705 cancer cell lines (left panel). The right panel displayed significant associations between growth inhibitory responses to cyclopamine and expressed HH pathway regulated species (right panel). (B) Cyclopamine-mediated growth responses in 20 small cell lung cancer (SCLC) cell lines. The 6 most growth inhibited lines are shown. (C) Cyclopamine-mediated growth inhibitory responses for 107 non-small cell lung cancer (NSCLC) lines. The 28 most growth inhibited lines are shown. Fractional growth responses for all panels (A, B and C) are displayed. Bold text indicated the most cyclopamine-responsive cells.

A total of 705 different human cancer lines were examined for responses to the Smo inhibitor cyclopamine (10 μM). Proliferation was reduced in a subset of cancer cell lines after 72 h of treatment (Fig. 1A, left panel). Of these lines, 353 were displayed in the Sanger database. No Ptch1 or Smo mutations were detected (3 lines had Smo amplifications that did not confer cyclopamine hypersensitivity). Data from the Sanger database showed the distribution of somatic mutations in Ptch1 for 860 unique cancer cell lines. Six had variant sequences of uncertain functional consequences. For the Smo gene, only 6 out of 866 different cancer cells had sequence variants of uncertain functional consequence. There were 152 lung cancer cell lines analyzed. None of these lines had Ptch1 or Smo mutations.

The HH pathway can affect expression of the Gli family members, Ptch1, cyclin D1, cyclin E, IGF2, IGFBP6, or GILZ (1–5). Cyclopamine-dependent growth responses were compared to these expressed species using the described database. Most highly significant associations occurred for high cyclin E (P=0.000009) and low IGFBP6 (P=0.000004) levels (Fig. 1A). Less significant associations were detected for IGF2 (P=0.005), cyclin D1 (P=0.024), Gli1 (P=0.04), and Gli2 (P=0.05). High GILZ levels were linked to reduced cyclopamine response (P= 0.002).

**Figure 2.**
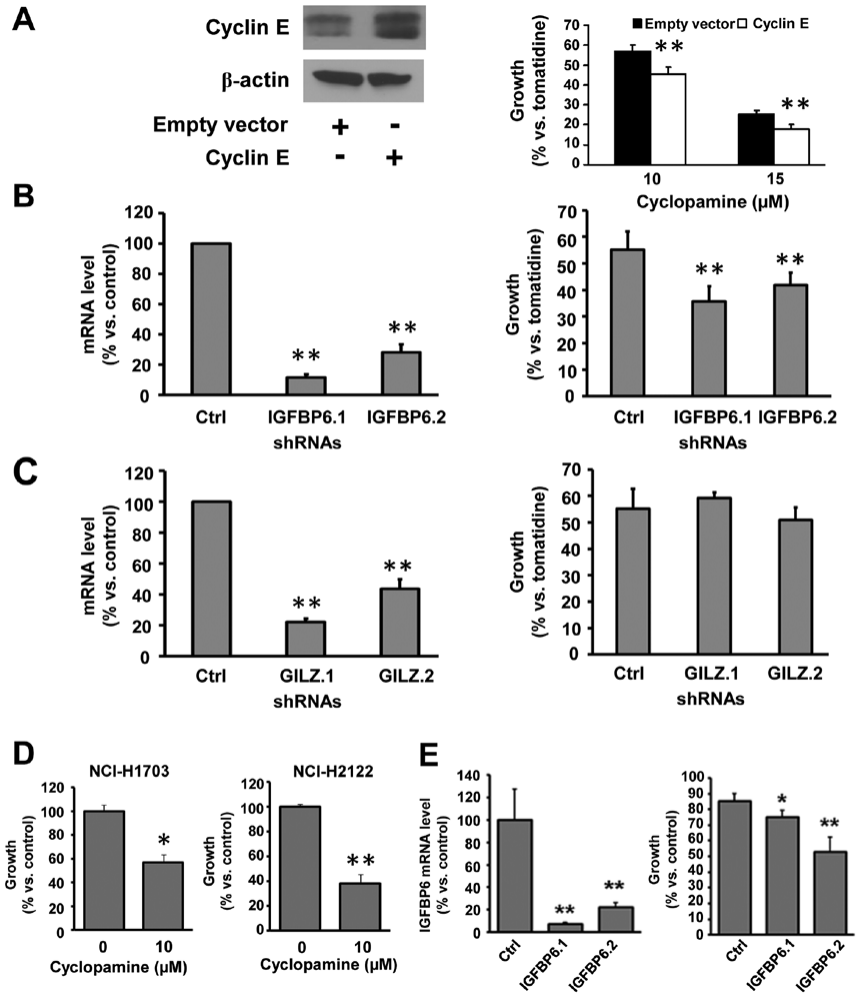
Functional validation of species associated with Smo-mediated growth inhibition. (A) Cyclin E was overexpressed in C-10 murine immortalized lung epithelial cells (left panel). Cyclin E overexpression enhanced response to cyclopamine treatment versus tomatidine controls (right panel). (B) IGFBP6 knock-down in ED-1 lung cancer cells (left panel) increased response to cyclopamine treatment versus controls (ctrl) (right panel). (C) GILZ knock-down in ED-1 cancer cells (left panel) did not significantly affect cyclopamine response (right panel). (D) Independent treatments of the human lung cancer cell lines NCI-H1703 (left panel) and NCI-H2122 (right panel) with cyclopamine (10 μM) for three days reduced cell growth versus vehicle control. Basal IGFBP6 levels were at the lowest limit of detection by real-time PCR assays in NCI-H1703 cells (data not shown), consistent with the enhanced sensitivity of these cells to cyclopamine. (E) Higher IGFBP6 levels were detected in NCI-H2122 relative to NCI-H1703 cells. When IGFBP6 expression was knocked-down by transfection of two independent siRNAs (left panel), the sensitivity of NCI-H2122 to cyclopamine (10 μM) was enhanced (right panel). Standard deviation bars are shown. ^*^P<0.05; ^**^P<0.01.

Twenty lines were of SCLC and 107 were of NSCLC origins. Subsets of SCLC (Fig. 1B) and NSCLC (Fig. 1C) lines responded to cyclopamine with substantial growth inhibition. NSCLC responses occurred independently of mutations for Ptch1 or Smo (Table IA). These responses were also independent of ras, epidermal growth factor receptor (*EGFR*), or *p53* mutations (Table IB). *K-RAS* mutations in NSCLC are associated with resistance to EGFR-tyrosine kinase inhibitors (TKIs) (32–35). Growth inhibition in the most cyclopamine-responsive NSCLC cells lines (NCI-H1703 and NCI-H2122) were independently confirmed in Fig. 2.

Studies were next conducted in lung cancer because an autocrine HH pathway is reported in lung cancer (36). HH signaling is important for growth of NSCLC and SCLC (6,15). Other reasons for studying lung cancer included the finding that increased cyclin E expression was associated with cyclopamine response (Fig. 1A) and cyclin E transgenic mice recapitulated key features of human lung cancer biology (16,30).

Gain or loss of expression was achieved for the most significantly associated candidate regulators of cyclopamine response (cyclin E and IGFBP6) or resistance (GILZ). Gli1 is an established HH pathway regulator in lung cancer (15). To determine whether cyclin E affected cyclopamine response, cyclin E was overexpressed in murine immortalized C-10 lung epithelial cells (Fig. 2A, left panel shows a 2.26-fold increase in cyclin E protein versus actin expression) and this significantly (P<0.01) increased cyclopamine-mediated growth inhibition versus the inactive isomer tomatidine (Fig. 2A, right panel). Similar results were obtained using a second Smo inhibitor, SANT-1 (data not shown), which was consistent with prior studies (15).

Two shRNAs (Fig. 2B, left panel) were selected to reduce IGFBP6 expression independently in ED-1 lung cancer cells derived from a transgenic mouse that expressed wild-type cyclin E (30). Individual knock-down with these shRNAs significantly (P<0.01) increased Smo-mediated growth inhibition in this murine lung cancer cell line versus an inactive shRNA control (Fig. 2B, right panel). Thus, gain of cyclin E or loss of IGFBP6 expression regulated response to a Smo inhibitor. In contrast, two different shRNAs independently repressed GILZ expression (Fig. 2C, left panel), but these did not appreciably affect cyclopamine-mediated growth suppression at 72 h (Fig. 2C, right panel). Findings were confirmed in human NSCLC cells engineered with IGFBP6 knock-down (Fig. 2D and E).

**Figure 3.**
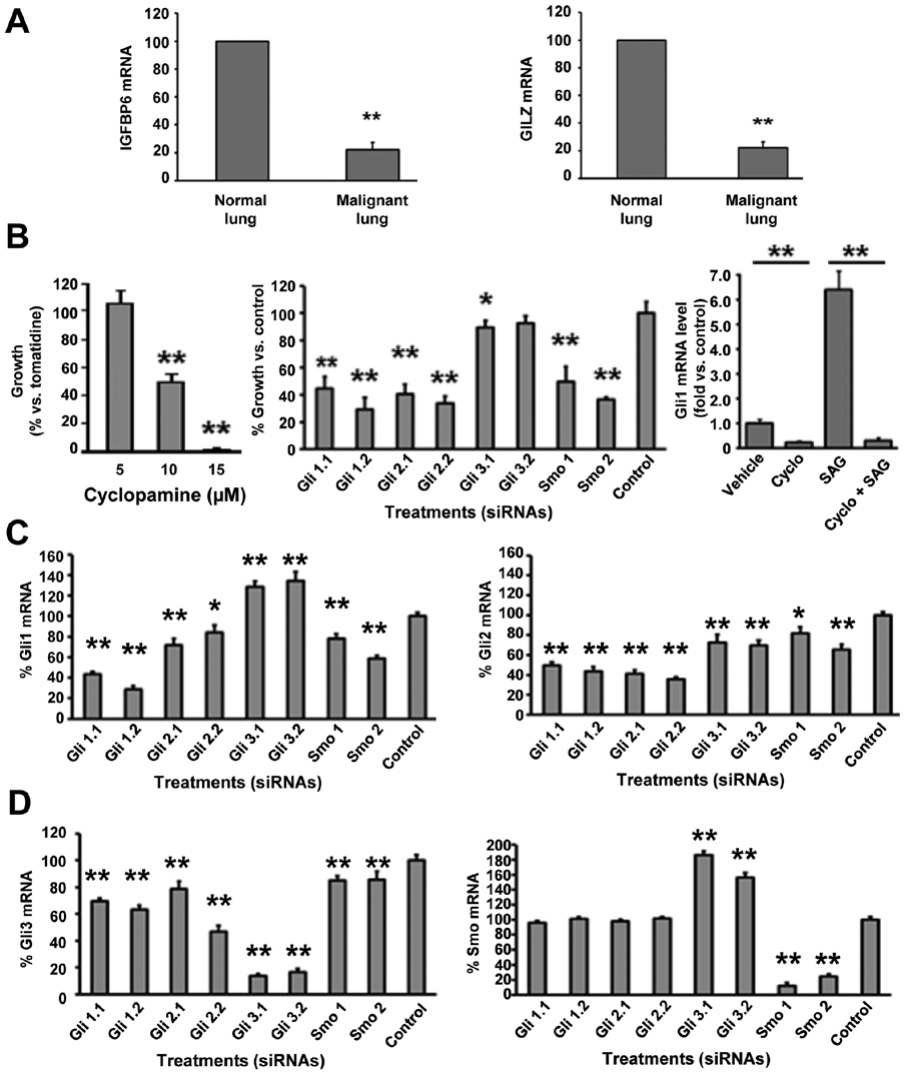
Hedgehog (HH) pathway member expression and regulation in lung cancers and lung cancer cell lines. (A) Cyclin E transgenic lung cancers expressed lower levels of IGFBP6 versus the adjacent normal lung tissue (left panel). Cyclin E transgenic lung cancers expressed lower mRNA levels of GILZ as compared to the adjacent normal lung tissue (right panel). These data are representative results of three triplicate replicate experiments. (B) ED-1 cells responded to pharmacologic (left panel) or siRNA-mediated (middle panel) repression of HH pathway members. Gli1 mRNA levels are shown 3 days after cyclopamine (cyclo, 10 μM), SAG (50 nM), or combined treatments (right panel). (C and D) Validation of individual Gli1, Gli2, Gli3, or Smo knock-downs in ED-1 cells. Compensatory effects on HH pathway members are shown. ^*^P<0.05; ^**^P<0.01. Standard deviation bars are displayed.

Lung cancers arising in transgenic mice from increased cyclin E expression in the lung exhibit high levels of Gli1 (16,30). These transgenic lung cancers are also shown to express low levels of IGFBP6 and GILZ versus the adjacent normal lung (Fig. 3A). Whether cell lines derived from these murine lung cancers responded to a Smo inhibitor was independently studied in ED-1 and ED-2 lung cancer cells derived respectively from transgenic mice whose lung tumors expressed wild-type (ED-1) or degradation-resistant (ED-2) cyclin E species (30).

Effects of Smo inhibition were explored by targeting Smo in ED-1 cells. Gli1, Gli2, Gli3, and Smo were independently targeted using two different siRNAs engineered to repress each of these species. Independent knock-down of Gli1, Gli2, and Smo each significantly inhibited ED-1 cell growth (as did cyclopamine treatment), but Gli3 knock-down did not appreciably affect growth (Fig. 3B, left and middle panels). As expected, the Smo agonist SAG augmented Gli1 expression; cyclopamine co-treatment prevented this (Fig. 3B, right panel). Compensatory changes in expressed HH pathway members were found after these different knock-downs in Fig. 3C and D. Smo knock-down substantially decreased lung cancer cell growth.

**Figure 4.**
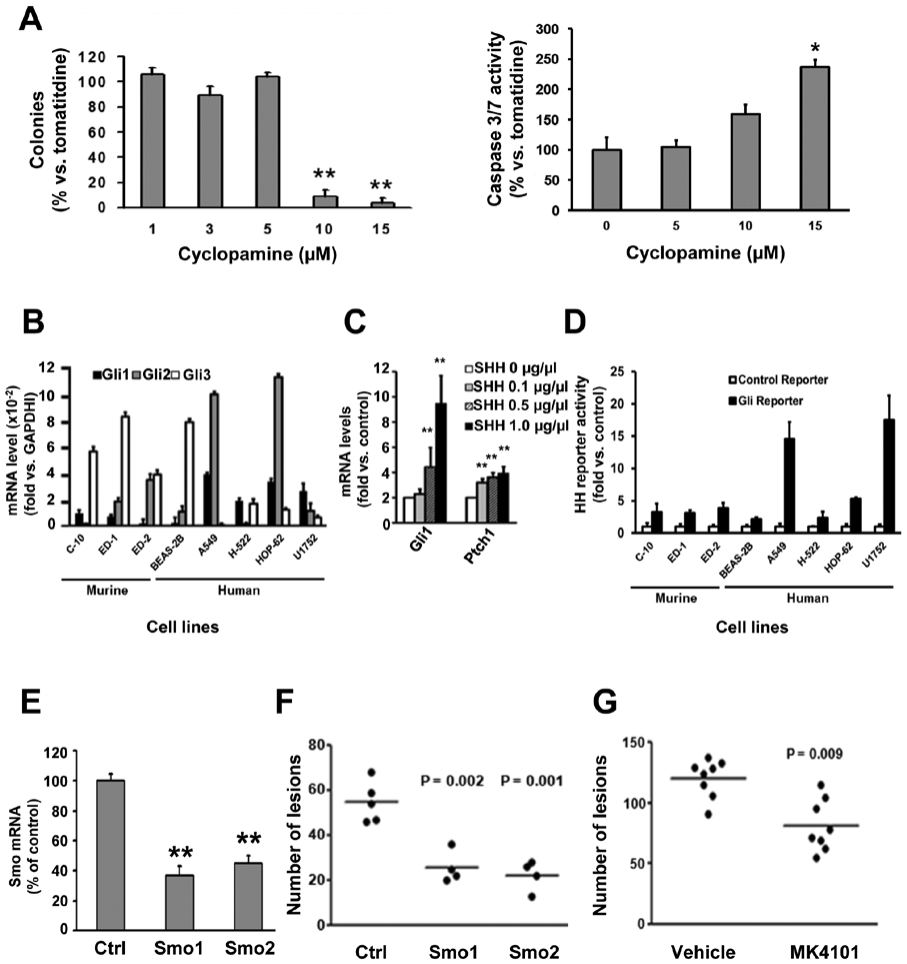
Expression of HH pathway members in immortalized pulmonary epithelial and lung cancer cell lines. Effects of Smo inhibition on lung cancer cell clonal growth, apoptosis and lung tumor formation were also examined. (A) Cyclopamine treatment of ED-1 cells significantly inhibited clonal growth of this lung cancer cell line (left panel). This treatment also increased apoptosis as scored by caspase 3/7 activity (right panel). In these panels, dose-responsive effects are displayed with comparisons made to tomatidine treatments. (B) ED-1 and ED-2 lung cancer cells expressed multiple HH pathway members. Findings were compared to C-10 immortalized murine lung epithelial cells. Analogous experiments were performed using BEAS-2B immortalized human bronchial epithelial cells and human lung cancer cell lines. The mRNA levels are shown. (C) Recombinant sonic hedgehog (sHH) significantly induced mRNA levels of the indicated HH regulated species in ED-1 cells. (D) Gli-BS-luciferase reporter activity in murine and human immortalized lung epithelial and lung cancer lines. (E) Repression of Smo by independent transfection of shRNAs (Smo1 and Smo2) versus controls. (F) Significant reduction of lung cancer formation *in vivo* after transplantation of ED-1 cells that express Smo1 or Smo2 shRNAs versus control shRNA. Each circle represents an individual mouse; horizontal lines indicate mean tumor numbers. (G) *Ex vivo* treatment of ED-1 cells with the Smo inhibitor MK-4101 before tail vein injection into syngeneic mice also reduced lung tumor formation. Each circle represents an individual mouse; horizontal lines indicate mean tumor numbers. Standard deviation bars are shown. ^*^P<0.05; ^**^P<0.01.

Cyclopamine treatment also significantly (P<0.01) decreased ED-1 clonal growth while increasing apoptosis (Fig. 4A). ED-1 and ED-2 cells expressed multiple HH pathway components as did the examined murine and human immortalized pulmonary epithelial or cancer cell lines (Fig. 4B). Genomic DNA from each of these cell lines was sequenced for the entire coding region of Ptch1 and for Smo mutations in regions that conferred Smo inhibitor resistance (A324T, V404M, D473H, E518K, W535L, and T640A). No mutations were detected (data not shown). Human lung cancer cell lines studied were those in which responses to Smo inhibition were already reported (15,36). To establish whether the HH pathway was functional in ED-1 cells, these cells were treated with recombinant sHH at different dosages in low serum (0.5%) containing medium. HH pathway activity in these cells was confirmed; sHH treatment significantly (P<0.01) increased mRNA expression for Gli1 and Ptch (Fig. 4C). Although ED-1 cells arose from transgenic lung cancers that activated the HH pathway (16), these cells had relatively low basal Gli1 and Gli2 mRNA levels (Fig. 4B) and low basal Gli1-reporter activity (Fig. 4D). This is consistent with differences reported between *ex vivo* versus *in vivo* HH pathway dependence of lung cancer cells (37). It was therefore not unexpected to detect prominent Gli1 immunostaining of lung tumors arising from ED-1 cells after their tail-vein injections into syngeneic FVB mice (data not shown).

To explore independently Smo inhibitor effects on lung cancer cells, ED-1 cells were engineered to have Smo knockdown. This was done using two independent shRNAs versus an inactive control shRNA (Fig. 4E). This conferred significant repression (Smo1 shRNA P=0.002 and Smo2 shRNA P=0.001) of transplanted lung cancers (Fig. 4F). *Ex vivo* treatment of ED-1 cells with another Smo inhibitor MK-4101 significantly reduced ED-1 colony formation (P<0.01, data not shown) and tumorigenicity (P=0.009) after tail-vein injections into syngeneic mice of these versus control cells (Fig. 4G).

**Figure 5.**
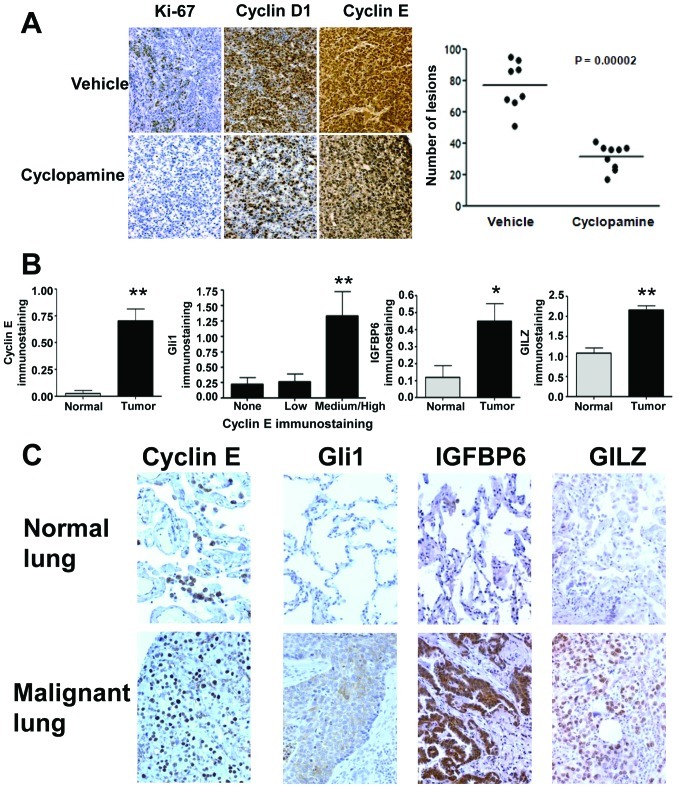
Cyclopamine treatment reduced proliferation and lung tumor formation in transgenic cyclin E mouse models. The expression profiles of HH pathway members in paired human malignant-normal lung tissues are displayed. (A) Decreased immunohistochemical detection of Ki-67, cyclin D1 and cyclin E in representative lung adenocarcinomas of transgenic cyclin E mice treated with cyclopamine versus vehicle controls (left panel). Cyclopamine significantly (P=0.00002) decreased lung cancers in the described murine syngeneic transplantable lung cancer model (right panel). Each circle represents an individual mouse; horizontal lines display mean tumor numbers. (B) Cyclin E, IGFBP6, and GILZ were each significantly (P<0.01) differentially expressed in human lung cancer versus normal lung. Cancers with high cyclin E immunostaining expressed high Gli1 levels (P<0.01). (C) Individual immunohistochemical expression for cyclin E, Gli1, IGFBP6, and GILZ in representative human normal versus malignant lung tissues. ^*^P<0.05; ^**^P<0.01.

Cyclopamine responses were examined in cyclin E transgenic mice because their lung cancers expressed a gene profile that was indicative of dependence on HH signaling. Cyclopamine-treatment decreased immunohistochemical expression of the proliferation markers Ki-67, cyclin D1 and cyclin E in the neoplastic versus normal lungs of treated versus age and sex-matched control mice (Fig. 5A, left panel). This result suggested that Smo inhibition would also reduce lung tumor formation in the transplantation model. ED-1 cells were injected into the tail veins of syngeneic mice. Cyclopamine treatment of mice began after lung tumors were histologically present (data not shown). This significantly (P=0.00002) reduced lung cancers in cyclopamine versus vehicle-treated mice (Fig. 5A, right panel).

To learn whether a profile indicative of HH pathway dependence was expressed in human lung cancers, a normal-malignant lung tissue array (20) was studied. Immunohistochemical expression profiles revealed significant differences in the malignant versus normal lung for cyclin E (P<0.01), IGFBP6 (P<0.01), and GILZ (P<0.01) in Fig. 5B. Lung cancers with high cyclin E levels expressed significantly (P<0.01) higher levels of Gli1 than did cases with reduced cyclin E levels (Fig. 5B). Cases with high cyclin E and low IGFBP6 levels had increased (P<0.0001) Gli1 immunohistochemical expression. Representative results are presented (Fig. 5C).

## Discussion

Smo inhibitors are active against Gorlin syndrome-associated BCC or medulloblastoma where Ptch mutations occur (9–13). Smo mutations confer resistance to Smo inhibitors (17,18). This study comprehensively interrogated 705 epithelial cancer cell lines for growth response to the Smo inhibitor cyclopamine. Findings were compared with expressed HH pathway-regulated species using a linked genetic database. Ptch and Smo mutations that respectively conferred Smo inhibitor response or resistance were undetected. Rare variant sequences were found, but their functional impact was not established. Because HH pathway activation occurs in lung cancers, findings were validated using different Smo inhibitors in human lung cancer cell lines, transgenic and transplantable murine lung cancer models, and paired normal-malignant lung tissue arrays. Ptch1 or Smo mutations were undetected in examined murine and human immortalized lung epithelial and cancer cell lines.

Smo inhibitor growth response was most significantly associated with high cyclin E (P= 0.000009), low IGFBP6 (P=0.000004), and high Gli1 (P=0.04) levels; high GILZ levels were associated with reduced response (P= 0.002) in Fig. 1. This profile implicated a basal dependence on the HH pathway for the growth of these cancer cells. This possibility was validated in murine and human lung cancer cell lines and transgenic as well as transplantable murine lung cancer models in Figs. 1–5. Functional consequences were shown using several pharmacological and genetic Smo inhibitors.

Differential expression of the same species was observed in a malignant versus normal human lung tissue array (Fig. 5). A profile indicative of Smo inhibitor response was also observed in murine lung cancers (Fig. 3). While high IGFBP6 levels were present in some lung tumors, it is notable that lung cancers with both high cyclin E and reduced IGFBP6 expression significantly (P<0.0001) increased Gli1 expression. This pattern was one indicating response to a Smo inhibitor. This points out the potential clinical need to discern profiles of HH pathway-regulated species in human tumors to learn which are likely to be responsive to a Smo inhibitor. Future clinical trials that explore activity of Smo inhibitors should also determine these profiles to uncover possible HH pathway dependence.

Several Smo inhibitors conferred similar effects. Stromal effects are engaged to confer some of these anti-neoplastic effects. This could account for differences between *in vitro* and *in vivo* effects of Smo inhibition (37,38). The findings presented in Fig. 1 could explain why trials with Smo inhibitors might underestimate clinical anti-tumor effects of Smo inhibition. Only some cancers express a gene profile indicating possible HH pathway dependence. Notably, autocrine HH pathway signaling occurs in lung cancers (36). The findings reported here are consistent with this prior study.

These findings have implications for combination cancer therapy. Responses to Smo inhibition occurred whether or not *RAS* or *p53* (or *Ptch1* or *Smo*) mutations were present in cancer cells (Table I). In clinical lung cancers, *K-RAS* mutations confer resistance to EGFR-TKIs (32,34,35). Yet, clinical trials revealed activity against lung cancers having *K-RAS* mutations when an EGFR-TKI was combined with a rexinoid (33). This clinical activity was associated with reduced cyclin D1 expression in post-treatment lung cancer biopsies (33). In the present study, *in vivo* responses to Smo inhibition were linked to cyclin D1 repression (Fig. 5). Adding a Smo inhibitor to a regimen that targets cyclin D1 for repression might enhance clinical anti-tumor activity. Smo responses were also associated with cyclin E expression (Figs. 1 and 5). Targeting the cyclin E-cdk2 complex exerted anti-tumor responses despite presence of *K-RAS* mutations by inducing anaphase catastrophe (19,39). Combining a cdk2 antagonist with a Smo inhibitor might augment anti-neoplastic activity.

High Smo inhibitor dosages were associated with these anti-neoplastic effects. A similar dose-response relationship for HH inhibition was observed in different tumor contexts and this might depend on expressed drug transporters (40,41). It is notable that the findings displayed in Fig. 3B (right panel) using a Smo agonist argue against off-target effects of cyclopamine. The reduction of lung tumors after Smo inhibition in the transplantation model reported here is notable since these lung cancers did not have Ptch1 or Smo mutations. Smo inhibitors might treat or prevent other cancers that lack these mutations.

Taken together, findings presented here indicate a Smo inhibitor should be considered in cancers that lack Smo or Ptch1 mutations. This is especially the case when the tumors express a gene profile indicating basal activation of the HH pathway. This could implicate a dependence on the HH pathway for growth or survival of the same tumors. Future clinical research should explore the translational consequences of these findings for cancer therapy and prevention.

## Figures and Tables

**Table I.** The (A) Ptch1 and Smo mutation status and (B) ras, p53, and activating EGFR mutation status of the 20 most cyclopamine-sensitive NSCLC cell lines examined in this study.

A, Ptch1 and Smo mutation status		
Cell line	Ptch1 status	Smo status
NCI-H2122	Wild-type	Wild-type
NCI-H1703	Wild-type	Wild-type
SW900	Wild-type	Wild-type
PC-9	Not known	Not known
NCI-H1299	Wild-type	Wild-type
Calu-1	Wild-type	Wild-type
NCI-H1944	Not known	Not known
NCI-H2172	Not known	Not known
NCI-H3122	Not known	Not known
201T	Not known	Not known
NCI-H1573	Wild-type	Wild-type
COR-L23	Wild-type	Wild-type
NCI-H2170	Wild-type	Wild-type
LU65B	Not known	Not known
NCI-H611	Wild-type	Wild-type
LU65A	Not known	Not known
NCI-H650	Wild-type	Wild-type
NCI-H322	Wild-type	Wild-type
RERF-LC-Sq1	Not known	Not known
NCI-H2347	Wild-type	Wild-type

B, ras, p53, and activating EGFR mutation status			
Cell line	Ras status	EGFR status	p53 status
NCI-H2122	Mutant (K-RAS)	Wild-type	Mutant
NCI-H1703	Wild-type	Wild-type	Mutant
SW900	Mutant (K-RAS)	Wild-type	Mutant
PC-9	Wild-type	Mutant	Mutant
NCI-H1299	Mutant (N-RAS)	Wild-type	Wild-type
Calu-1	Mutant (K-RAS)	Wild-type	Wild-type
NCI-H1944	Mutant (K-RAS)	Not known	Wild-type
NCI-H2172	Not known	Not known	Not known
NCI-H3122	Not known	Wild-type	Not known
201T	Wild-type	Wild-type	Not known
NCI-H1573	Mutant (K-RAS)	Wild-type	Mutant
COR-L23	Mutant (K-RAS)	Wild-type	Wild-type
NCI-H2170	Wild-type	Wild-type	Mutant
LU65B	Not known	Not known	Mutant
NCI-H611	Wild-type	Wild-type	Mutant
LU65A	Not known	Not known	Mutant
NCI-H650	Mutant (K-RAS)	Wild-type	Mutant
NCI-H322	Wild-type	Wild-type	Mutant
RERF-LC-Sq1	Not known	Wild-type	Not known
NCI-H2347	Mutant (N-RAS)	Wild-type	Wild-type
